# Evaluation of contrast wash-in and peak enhancement in adenosine first pass perfusion CMR in patients post bypass surgery

**DOI:** 10.1186/1532-429X-12-28

**Published:** 2010-05-13

**Authors:** Sebastian Kelle, Kristof Graf, Stefan Dreysse, Bernhard Schnackenburg, Eckart Fleck, Christoph Klein

**Affiliations:** 1 German Heart Institute Berlin, Germany; 2 Philips Clinical Sciences, Hamburg, Germany

## Abstract

**Background:**

Adenosine first pass perfusion cardiovascular magnetic resonance (CMR) yields excellent results for the detection of significant coronary artery disease (CAD). In patients with coronary artery bypass grafts (CABG) the kinetics of a contrast bolus may by altered only due to different distances through the bypass grafts compared to native vessels, thereby possibly imitating a perfusion defect. The aim of the study was to evaluate semiquantitative perfusion parameters in order to assess possible differences in epicardial contrast kinetics in areas supplied by native coronaries and CABG, both without significant stenosis.

**Methods:**

Twenty patients with invasive exclusion of significant CAD (control group) and 38 patients with CABG without angiographically significant (≥50%) stenosis in unbypassed coronaries or grafts were retrospectively included in the study. They underwent adenosine first pass (0.05 mmol/kg Gd-DTPA) perfusion (3 short axis views/heart beat) and late gadolinium enhancement (LGE) imaging 1 day before invasive coronary angiography. Areas perfused by native coronaries and/or the different bypasses were identified in X-ray angiography using the 16 segment model. In each of these areas upslope and maximal signal intensity (SI_max_) relative to the left ventricular parameters, time to 50% maximal signal intensity (T_SI50%max_) and time to maximal signal intensity (T_SImax_) were calculated.

**Results:**

In areas perfused by coronary arteries with bypasses compared to native coronaries relative upslope and relative SI_max _did not show a significant difference. T_SI50%max _and T_SImax _in native coronaries and bypasses were 7.2s ± 1.9s vs. 7.5s ± 1.9s (p < 0.05) and 12.6s ± 3.0s vs. 13.1s ± 3.0s (p < 0.05), respectively. The delay in T_max _resulted in a significant (p < 0.05) delay of 0.5 ± 1.1 heart beats (=images) when adjusted to the heart rate. Differences in time were most pronounced in areas perfused by left internal mammary artery grafts rather than by venous CABG, but were also present between native vessel territories in patients without CAD, albeit with smaller variability.

**Conclusion:**

Adenosine perfusion CMR in patients post CABG may be associated with a short delay in contrast arrival. However, once the contrast is in the myocardium there is similar wash-in kinetics and peak enhancement. Therefore, since the delay is only short, the possibly differing contrast kinetics through grafts and native vessels does not seem to be a limiting factor for the accuracy of first pass adenosine perfusion in patients post CABG.

## Background

Cardiovascular magnetic resonance (CMR) is an accurate diagnostic tool for the detection and characterization of coronary artery disease (CAD). It offers both functional studies for the detection of ischemia and tissue characterization for the detection and quantification of myocardial infarction. Multiple trials have demonstrated high diagnostic accuracy of first pass adenosine perfusion [1-6] with potential advantages (e.g. higher spatial resolution) compared to nuclear imaging. However, only two studies have evaluated this technique in patients post coronary arterial bypass grafting (CABG); these demonstrated good sensitivity and specificity [7,8]. Myocardial blood flow may be more complex after surgery, mainly because of more severe disease, incomplete revascularization and/or the presence of myocardial infarction. Additionally first pass kinetics of a contrast bolus may be altered due to the different distances to the myocardial territories through native vessels compared to those with bypasses. Thus, diagnostic accuracy of adenosine stress perfusion CMR may be reduced, especially by possible mimicking of perfusion defects. Due to their ease of performance, most perfusion CMR studies are analyzed visually taking into account the contrast arrival, contrast wash-in kinetics and maximal signal intensity (SI_max_) of peak myocardial enhancement.

The aim of the study was therefore to evaluate the contrast wash-in kinetics, represented by the semiquantitative perfusion parameters upslope of the signal intensity time curves, time to SI_max _(T_SImax_), time to 50% of SI_max _(T_SI50%max_) and peak enhancement/maximal signal intensity (SI_max_) in patients after CABG in order to evaluate contrast kinetics in areas supplied by native coronaries and different bypass grafts.

## Methods

The study was approved by the Institutional Review Board of the Charité, Berlin, Germany. Eighty-nine patients with status post CABG who had both adenosine first pass perfusion study and coronary X-ray angiography were retrospectively analyzed. The coronary angiography was indicated for clinical reasons and was performed independently of the result of the perfusion study. To reduce the influence of steal effects and/or competitive flow on contrast kinetics and therefore possible alteration of the semiquantitative parameters in different vessel territories, patients were excluded if a significant stenosis (≥50% luminal narrowing) in grafts or unbypassed native vessels ≥2 mm in diameter was detected. Fifty-one patients fulfilled these exclusion criteria; therefore 38 patients formed the final study group.

Mean time between surgery and CMR was 7.4 ± 4.5 years (range 1-21 years). Meanwhile a coronary intervention had been performed in 20 patients (8 in coronary arteries, 4 in bypass grafts and 8 in both). Mean time between surgery and intervention was 5.3 ± 3.9 years and between intervention and CMR 2.0 ± 1.9 years (range 0.2-7.3 years). To evaluate a normal range and variety of the semiquantitative parameters that may occur in native vessel territories within one patient, these were calculated in patients with an identical imaging protocol, but invasive exclusion of significant CAD.

### Cardiovascular magnetic resonance

All patients were examined in supine position using a 1.5 Tesla scanner (Intera, Philips Medical Systems, Netherlands). A five-element cardiac synergy coil was used for signal detection. A rapid gradient echo sequence allowed localization of the heart in the three standard planes. The study protocol consisted of first pass stress perfusion (SSFP, TE/TR/flip angle 2.7/1.4/50°, spatial resolution 2.8 × 2.9 × 8.0 mm^3^, acquisition time 144 ms, 1 saturation prepulse per slice, prepulse delay 100 ms, 3 slices/heart beat) 3-4 minutes after adenosine infusion (140 μg/min/kg body weight) using a peripheral contrast bolus of 0.05 mmol/kg body weight (Gd-DTPA: Magnevist, Schering, Germany) during breath holding. For the detection of myocardial scar the left ventricle (LV) was imaged in short axis and the standard long axis views 10 minutes after administration of an additional contrast bolus (0.15 mmol/kg) using an inversion recovery 3D-turbo gradient echo technique (TE/TR/flip angle 2.3/4.8/15°, spatial resolution 1.4 × 1.4 × 5.0 mm^3^, acquisition time 170 ms, prepulse delay 225 - 300 ms).

#### Image Analysis

Regional perfusion was evaluated in the 3 standard short-axis slices (apical, middle and basal) using the 16-segment model [9]. The endo- and epicardial borders of the three short axis views were defined and copied to all dynamics. Manual adjustments were made whenever movements occurred to ensure myocardial enhancement only. Semiquantitative perfusion analysis was performed with a specialized software package (ViewForum, Philips Medical Systems, The Netherlands).

**Figure 1:**
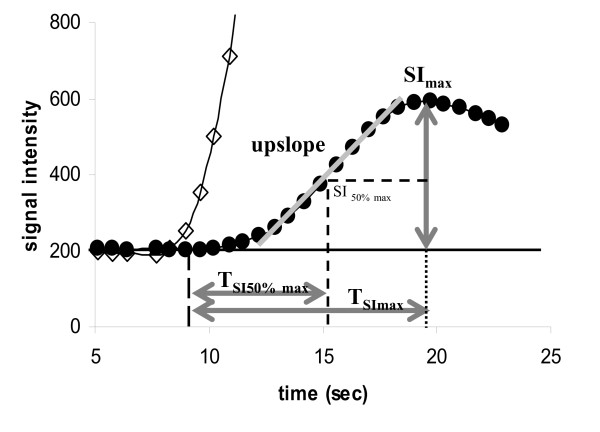
**Analysis of the signal intensity (SI) curves. The left ventricular signal intensity curve (squares) is cut on the top**. Upslope and SI_max _were assessed in (%) compared to the LV values of the medial short axis slice, T_SI50%max _and T_SImax _in seconds.

Mean SI before contrast agent injection was subtracted from all post contrast data. The myocardial and LV upslopes of the resulting SI time curves were determined using a linear fit of 5 consecutive images in myocardial curves (3 in the LV curves to account for the shorter bolus duration in the LV versus the myocardium). Maximal SI was defined as the maximal signal intensity in the LV and the myocardial segments. Upslope and maximal signal intensity (SI_max_) of the myocardium and LV were placed in relation to each other, calculating the relative SI_max _(in % compared to the LV values of the middle short axis) and the relative upslope. Time (seconds) measured from the contrast arrival in the left ventricle to the maximum signal intensity (T_SImax_) and the time to 50% maximum signal intensity (T_SI50%max_) during the first pass of contrast in each myocardial segment (figure 1) was calculated. In order to analyze possible differences in the semiquantitative parameters on an epicardial level and to exclude differences on the myocardial level as far as possible, segments with any degree of late gadolinium enhancement (LGE) were excluded from the analysis. All selective coronary X-ray angiographies were performed within 24 hours after the CMR examination. Two experienced interventional cardiologists blinded to the results of the CMR examinations visually evaluated the angiograms.

**Figure 2:**
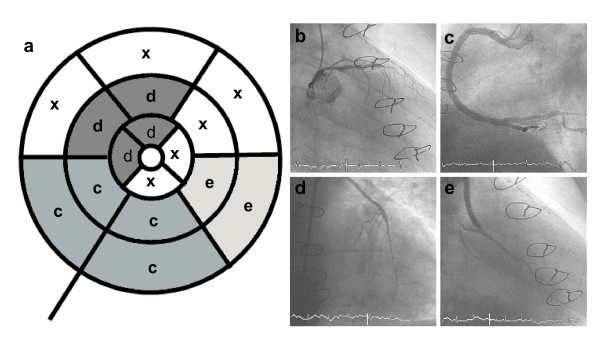
**Example of the selection of the perfusion territories as retrospectively defined by angiography**. On the left side (a) the polar map (three short axis slices, 16 segment model) with the segments included in the analysis according to angiography. The letters on the polar map correspond to those on the angiographic images. Segments marked with an "x" were not used for analysis, as care was taken to include segments with a very high probability of being supplied by the vessel/graft only. The proximal LAD (b) was not included for analysis, as the basal short axis view is planned to exclude the left ventricular outflow tract and may therefore not include the very basal anterior wall. The anterolateral segments (apical and medial) were not included due to the lack of visible distal diagonal branches. There is a large right coronary artery without significant stenosis (c), the LIMA graft on the distal LAD (d) and a venous bypass on a marginal branch (e).

As there is a wide variety in the perfusion territories of the coronary arteries, especially after CABG, we did not use the standard perfusion territories suggested by the AHA [9]. Therefore, in invasive angiography perfusion territories of the native coronaries and the bypass grafts using the 16 segment model were defined individually. Care was taken to try to include only the core segments of a perfusion territory of a vessel/graft and to exclude any segment that was not definitely perfused by the specific vessel/graft (figure 2). If a patient had two native coronary arteries without a bypass, the one with the larger perfusion territory was chosen for the comparison with the grafts. In the patients without CAD the perfusion areas of the three coronary arteries were compared, again as defined by X-ray angiography. In order to evaluate similar segments in the patients without CAD as in patients post CABG, distal perfusion areas were chosen, e.g. apical and medial segments for the left anterior descending (LAD) territory. The semiquantitative parameters of several segments in the previously defined perfusion area of a vessel/graft were then averaged and compared to each other (native vessels vs. all CABG, native vessels vs. left internal mammary artery graft (LIMA) and venous CABG separately, LIMA graft vs. venous CABG; additionally, LAD vs. left circumflex (LCX) vs. right coronary artery (RCA) in patients without CAD).

**Figure 3:**
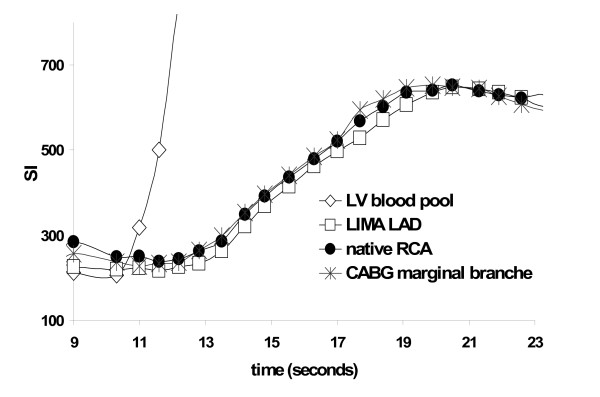
**Example of signal intensity time curve during adenosine vasodilatation of a native RCA, LIMA LAD and venous CABG to a marginal branch in one patient**.

The differences between these parameters in patients post CABG were calculated such that the lower the upslope or the SI_max _and the longer the T_SI50%max _or T_SImax _of CABG compared to native vessels, the more negative the result. These results were additionally divided to consider grafts bypassing totally occluded or stenotic vessels, as the kinetics of the contrast bolus may be altered if it passes through a stenotic native vessel in addition to a functioning graft rather than through the graft only. To evaluate whether not one parameter alone, but the combination of SI_max_, T_SI50%max _and upslope will be significantly altered and thus add up in areas perfused by grafts compared to native vessels an "add-up score" on a patient basis was calculated by the sum of

Thus, the lower the upslope, the longer the T_SI50%max _and the lower the SI_max _in areas perfused by grafts, the more negative the score will be. As the temporal resolution of adenosine stress perfusion is dependent on the heart rate (acquisition of 3 slices to every heart beat) the number of images of a possible delay in reaching SI_max_, the differences in T_SImax _coronaries and different grafts were evaluated by relating them to the heart rate during adenosine stress.

#### Statistics

Statistical analysis was performed using SPSS 17.0 for Windows (SPSS Inc.). For all continuous parameters means and standard deviations are given. For comparison of the parameters between perfusion areas within one patient population a nonparametric test (Wilcoxon) and between the two patient groups the Mann-Whitney test was used. Values < 0.05 were considered significant.

## Results

Twenty patients without CAD (three perfusion areas each) and 38 patients after CABG were included in the study. Eight grafts and 3 native coronary arteries were excluded from the analysis due to the presence of LGE, resulting in 29 native coronary (10 LAD, 10 LCX and 9 RCA) and 69 bypass territories (24 LIMA grafts to the LAD, 2 LIMA grafts to a diagonal branch, 8 venous bypasses to the LAD, 4 to a diagonal branch, 16 to a marginal branch and 15 to the RCA). A direct comparison within one patient was possible between native coronaries and 46 bypass grafts (20 LIMA grafts and 26 venous CABG) in 29 patients, between 26 native coronary arteries and venous grafts in 21 patients and between 20 LIMA grafts and 21 venous CABG in 14 patients. The average number of segments evaluated per patient was 9.9 ± 2.5, per native vessel 3.8 ± 1.5, per LIMA graft 4.8 ± 1.8 and per venous graft 3.0 ± 1.7. Patients' characteristics and CMR hemodynamics are shown in table 1.

**Table 1:** Patient characteristics and hemodynamics

	CABG (n = 38)	No CAD (n = 20)	p
**Men/women**	31/7	12/8	<0.05
**Age (years)**	64 ± 8 (48-78)	57 ± 12 (37-72)	n.s.
**Weight (kg)**	85 ± 12 (55-120)	76 ± 14 (55-100)	n.s.
**Body mass index**	28.2 ± 3.6 (21.3-37.6)	26.8 ± 4.1 (21.8-34.9)	n.s.
**Diabetes mellitus**	15 (39%)	5 (25%)	<0.05
**Hypertension**	29 (76%)	13 (65%)	n.s.
**Smoking**	14 (37%)	7 (35%)	n.s.
**Hypercholesterinemia**	35 (92%)	13 (65%)	n.s.
**LV ejection fraction**	53 ± 9% (26-71)	63 ± 5% (55-71)	<0.05
**LGE**	24 (63%)	0 (0%)	<0.05
**Heart rate rest (beats/min)**	65 ± 9	71 ± 11	n.s.
**Heart rate adenosine (beats/min)**	77 ± 11	87 ± 13	<0.05
**Blood pressure rest (mmHg)**	125 ± 20/70 ± 10	132 ± 18/72 ± 12	<0.05
**Blood pressure adenosine (mmHg)**	125 ± 20/67 ± 11	136 ± 18/76 ± 8	<0.05

**Figure 4:**
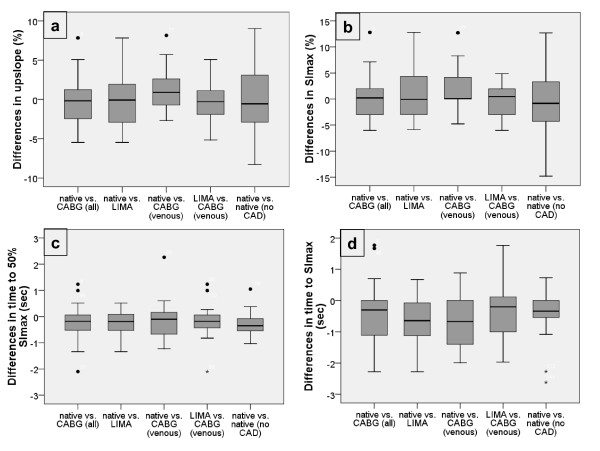
**Boxplot diagrams analyzing the differences in the upslope (a), maximal signal intensity (b), time to 50% of maximal signal intensity (c) and time to maximal signal intensity (d) between native vessels (native) and bypass grafts (CABG), also distinguishing between LIMA and venous grafts, as well as between native vessels in patients without CAD (native vs. native)**.

An example of the signal intensity time curves in one patient after CABG is shown in figure 3. Results of the semiquantitative parameters for native vessels vs. all CABG, native vessel vs. LIMA graft and vs. venous CABG and LIMA graft vs. venous CABG, as well as LAD vs. LCX vs. RCA in patients without CAD are shown in table 2. The differences in the parameters within each group are demonstrated in figure 4 (boxplot differences). No statistical difference was present for the upslope and SI_max_; however a small but statistically significant difference (p = 0.01) was found for T_SI50%max _and T_SImax _when comparing native vessels with all grafts or LIMA grafts, but not when comparing native vessels with venous grafts only. There was also a trend towards shorter arrival times in venous CABG compared to LIMA grafts. The difference between T_SImax _and T_SI50%max _when native vessels and CABG were compared did not demonstrate any statistical significance. In patients without CAD, upslope and SI_max _were significantly lower in the LCX compared to LAD and RCA and T_SI50%max _and T_SImax _significantly shorter in the LAD when compared to LCX and RCA (table 2).

**Table 2:** Semiquantitative perfusion parameters

		Relative SI_max _(%)	Relative Upslope (%)	T_50%SImax _(seconds)	T_50%SImax _(beats)	T_SIMax _(seconds)	T_SIMax _(beats)
***native/aCABG ****(n = 46)*	**native**	22.8 ± 8.2	18.3 ± 6.3	7.2 ± 1.9	9.4 ± 2.4	12.6 ± 3.0	16.3 ± 3.5
	**CABG**	22.9 ± 8.5	18.0 ± 6.5	7.5 ± 1.9*	9.6 ± 2.4*	13.1 ± 3.0*	16.8 ± 3.7*
***native/LIMA ****(n = 20)*	**native**	22.9 ± 8.7	18.2 ± 6.7	7.1 ± 2.0	8.9 ± 2.5	12.4 ± 3.1	15.7 ± 3.6
	**LIMA**	23.7 ± 10.6	18.0 ± 7.8	7.3 ± 1.9*	9.3 ± 2.3*	13.1 ± 3.0*	16.5 ± 3.4*
***native/vCABG ****(n = 26)*	**native**	22.6 ± 8.0	18.3 ± 6.1	7.4 ± 1.8	9.7 ± 2.4	12.8 ± 2.9	16.7 ± 3.4
	**CABG**	22.3 ± 6.6	18.0 ± 5.6	7.6 ± 2.0*	9.9 ± 2.5*	13.0 ±3.1	17.1 ± 3.9
***LIMA/vCABG ****(n = 21)*	**LIMA**	21.9 ± 9.4	17.4 ± 7.4	7.6 ± 1.8	10.2 ± 2.4	13.2 ± 2.9	17.7 ± 3.3
	**CABG**	20.1 ± 8.2	16.3 ± 6.3	7.4 ± 2.2	9.9 ± 2.6	12.5 ± 3.2*	16.8 ± 3.3*
***No CAD***							
***native/native ****(n = 20)*	**LAD**	31.7 ± 10.9	22.9 ± 4.7	5.7 ± 1.1*	7.6 ± 2.3*	11.1 ± 1.9*	14.4 ± 5.9*
	**LCX**	27.4 ± 9.2*	19.4 ± 4.2*	6.2 ± 1.2	8.3 ± 2.6	11.6 ± 2.1	15.0 ± 6.1
	**RCA**	32.8 ± 11.1	22.7 ± 5.0	6.1 ± 1.1	8.2 ± 2.5	11.6 ± 1.9	15.0 ± 6.0

* = p < 0.05; vCABG = venous CABG; aCABG = all CABG (LIMA and venous CABG)

**Figure 5:**
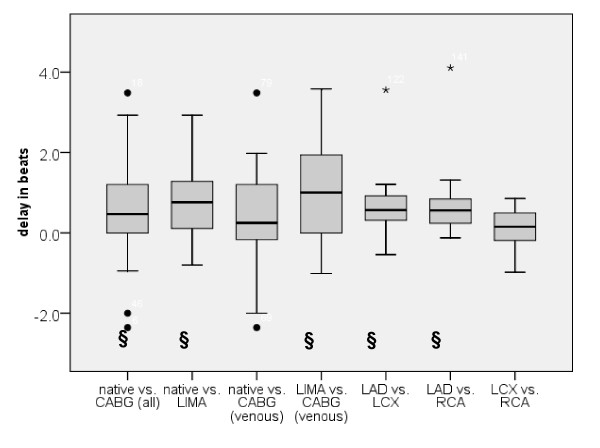
**Boxplot diagram analyzing the differences in the time to maximal signal intensity in heart beats between native vessels (native) and bypass grafts (CABG), also distinguishing between LIMA and venous grafts and between the three vessel territories in patients without CAD**. § = p < 0.05

**Figure 6:**
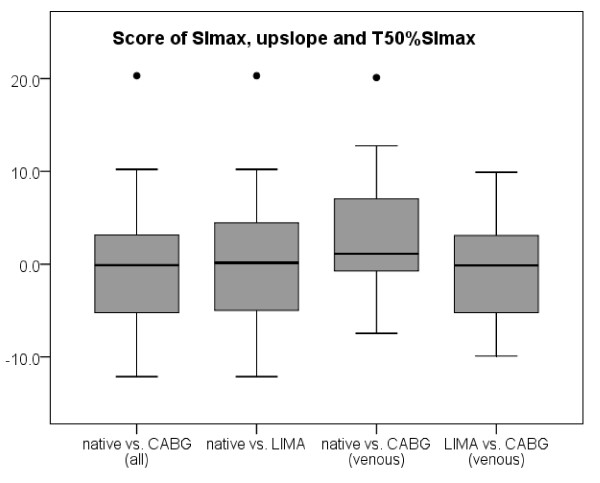
**Boxplot diagram analyzing the differences in the "add-up score" calculated from maximal signal intensity, upslope and time to 50% of the maximal signal intensity between native vessels (native) and bypass grafts (CABG), also distinguishing between LIMA and venous grafts**. There is no tendency towards negative results (the more negative the score the smaller the upslope, the longer the time to 50% of maximal signal intensity (T50%SImax) and the smaller the maximal signal intensity (SImax)); therefore no add-up effect of these parameters can be demonstrated.

An intrinsic delay of contrast arrival from the basal to the apical slice has been described. In our analysis, the LAD territory of patients without CAD was chosen, just like in the patients after CABG in the more apical segments. The RCA territory includes more basal segments, and no significant delay in the LAD compared to the RCA territory has been observed. Therefore, the longer T_SImax _of segments supplied by the LIMA graft does not seem to be due to the more apical location of the segments. The calculated delay to T_SImax _in heart beats (images) was 0.5 ± 1.1 for all CABG vs. native vessels (p = 0.002), 0.8 ± 1.0 for LIMA-graft vs. native vessels (p = 0.005), 0.4 ± 1.2 for venous vs. native vessels CABG (p = 0.14) and 0.9 ± 1.2 for LIMA-graft vs. venous CABG (p = 0.004) (fig. 5). However, statistical differences between LAD and LCX (0.7 ± 0.9) and RCA (0.7 ± 0.9) can also be demonstrated in patients without CAD (fig. 5). There is no significant (p > 0.05) difference in the "add-up score" of the combination of SI_max_, upslope and T_SI50%max _(fig. 6). If grafts supplying totally occluded vessels are differentiated from grafts supplying stenotic vessels there is a trend towards smaller upslopes and longer arrival times (table 3).

**Table 3:** Semiquatitative parameters (CABG supplying occluded vs. stenotic coronary arteries)

		Relative SI_max_(%)	Relative Upslope (%)	T_50%SImax _(seconds)	T_SIMax _(seconds)
***native vs all CABG ****(stenosed, n = 17)*	**native**	23.9 ± 9.3	19.0 ± 6.7	6.9 ± 1.4	12.0 ± 1.8
	**CABG**	24.9 ± 11.0	20.1 ± 7.7	7.1 ± 1.3	12.3 ± 1.8
***native vs all CABG ****(occluded, n = 29)*	**native**	22.1 ± 7.6	17.8 ± 6.1	7.4 ± 2.1	13.0 ± 3.5
	**CABG**	21.7 ± 6.6	16.7 ± 5.5*	7.7 ± 2.2*	13.5 ± 3.5*
					
***native vs LIMA ****(stenosed, n = 9)*	**native**	25.7 ± 11.1	20.0 ± 8.8	7.2 ± 1.8	12.4 ± 2.4
	**LIMA**	27.3 ± 14.3	21.2 ± 10.1	7.5 ± 1.6	13.0 ± 2.1*
***native vs LIMA ****(occluded, n = 11)*	**native**	20.1 ± 5.8	16.7 ± 4.2	6.9 ± 2.3	12.5 ± 3.8
	**LIMA**	20.8 ± 5.5	15.5 ± 4.0	7.2 ± 2.1	13.1 ± 3.6
					
***native vs venous CABG ****(stenosed, n = 8)*	**native**	21.9 ± 6.9	18.0 ± 3.4	6.5 ± 0.7	11.6 ± 0.9
	**CABG**	22.2 ± 5.2	19.0 ± 3.9	6.6 ± 0.7	11.6 ± 1.2
***native vs venous CABG ****(occluded, n = 18)*	**native**	23.0 ± 8.6	18.5 ± 7.0	7.7 ± 2.1	13.3 ± 3.3
	**CABG**	22.3 ± 7.3	17.5 ± 6.2§	8.0 ± 2.2*	13.7 ± 3.5

*: p < 0.05; §: p = 0.05

## Discussion

The present study demonstrates heterogeneous perfusion patterns between native vessels and different bypass grafts when using the first pass bolus technique, with, however, only small differences that are partly also present in different perfusion areas in patients without CAD. No significant difference in the peak enhancement (SI_max_) or myocardial contrast wash-in (upslope) exists between native coronaries and coronary arterial bypass grafts. However, contrast arrival expressed by the time from the appearance in the left ventricular cavity to peak myocardial enhancement or 50% of peak myocardial enhancement may be delayed, especially when grafts bypass occluded vessels. This, however, does not seem to be a limitation for adenosine perfusion in patients after CABG.

CABG is a common procedure for the treatment of significant CAD. Long-term graft patency and progression of CAD are the major factors limiting the initial clinical benefits of revascularization and patient survival. As exercise ECG has limitations (e.g. previous myocardial infarction and/or functional single-vessel disease), stress imaging tests are the preferred method for non-invasive testing in this subgroup [10]. There is little data on stress perfusion using adenosine [7,8,11], but it demonstrates reasonable diagnostic accuracy when compared to invasive angiography. However, diagnostic accuracy is reduced in patients with surgical revascularization [8]. The clinically most applicable analysis is the visual assessment of the contrast passage through the myocardium, as it is fast and yields good clinical results [12-14]. However, a reduction of peak enhancement, a delay in myocardial contrast arrival or a reduced speed of contrast wash-in purely by alteration of the contrast bolus due to bypass surgery may limit the use of the perfusion CMR technique.

Therefore we aimed to calculate these semiquantitative parameters to evaluate possible differences in first pass perfusion imaging in patients after bypass surgery depending on areas supplied by native vessels and vessels with arterial or venous bypasses. There are no direct comparisons between visual and semiquantitative analysis in the same patient population, as visual evaluation is believed to need higher contrast concentrations [4,14] and semiquantitative evaluation to need lower [5,15] concentrations, due to the loss of the relationship between signal intensity and contrast concentration, especially in the left ventricular cavity. For semiquantitative analysis the upslope is the most often used parameter [5,12] and has demonstrated good correlation compared to angiography and PET for the detection of significant CAD [16], even with the use of higher gadolinium concentrations [6]. In a direct comparison between upslope, maximal SI, time to maximal SI and contrast arrival time, the upslope was the best parameter for the detection of ischemia [17].

Although lacking the direct comparison between semiquantitative and visual analysis, we are certain that at least to a large extent parameters such as maximal signal intensity, time to maximal signal intensity and the speed of contrast wash-in (upslope) play a role in the visual assessment of first pass perfusion. In order to evaluate a normal range of these parameters in a perfusion study that was classified as non pathological, they were calculated in patients with exclusion of CAD by invasive X-ray angiography.

As demonstrated in table 2 and figures 4 and 5 there is a considerable intra-patient range of the semiquantitative parameters. The difference in delay in reaching peak enhancement is significant between the LAD and the two other vessel territories. However, this range seems to represent a normal variety of contrast kinetics during adenosine vasodilatation in uncompromised epicardial blood flow and is, most importantly, considered as normal by the evaluating physician with the contrast concentration used. Coronary flow reserve in bypass grafts is no different than in native vessels, except if supplying infarcted myocardium [18]. Therefore we took care not to include segments with LGE, known to represent myocardial infarction, in order to evaluate the semiquantitative parameters on the vessel/graft and not on the myocardial level.

There was no difference in maximal SI or upslope between native vessels and CABG, while there is a significant trend towards a delayed contrast arrival. There is the tendency that the delay of contrast arrival is more pronounced in LIMA than in venous grafts. This can be explained by the longer distance the bolus needs to travel to reach the myocardium. We did not use the time between contrast arrival in the LV and the myocardium, although this would represent the real arrival time. But, as the time is short compared to the temporal resolution (one heart beat) and therefore prone to miscalculation, especially as the exact start of myocardial enhancement is sometimes difficult to identify, we have decided against this parameter. As the upslope is similar T_SI50%max _and T_SImax _can be used as parameters for contrast arrival. Although the mean contrast delay in heart beats is below one beat (figure 5), there was a considerable deviation reaching up to almost 4 heart beats in one case. Therefore, similar to the visual impression, there are cases where areas perfused by grafts and especially by LIMA grafts may show a delay. However, the delay caused by the longer distance of the bolus to reach the myocardium should be transmural, while in true perfusion defects, hypoenhancement is non-transmural in the majority of cases [19]. As stated above, the semiquantitative parameters in patients without significant CAD also demonstrate significant differences in the different vessel territories. However, the differences in the number of heart beats needed to reach SI_max _demonstrate a smaller range than in patients after CABG (fig. 5). The overall larger upslopes/SI_max _and shorter T_SI50%max_/T_SImax _in patients without CAD compared to post CABG (table 2) are probably due to the fact that maximal blood flow in patients with CAD may be reduced even without significant stenosis, as well as due to lower ejection fraction and areas of hypo- and akinesia. Importantly, there is no evidence that the upslope and/or SI_max _are reduced as an add-up effect, if e.g. T_SI50%max _is prolonged in areas supplied by CABG, as there is no significant tendency in the positive or negative direction of the add-up score (fig. 6).

CMR perfusion patterns may possibly be altered by simultaneous blood flow, although probably reduced via a significantly stenosed native vessel in addition to the bypass graft, if the native vessel is not occluded. Therefore, we carried out a separate analysis of areas supplied by the graft only (occluded native vessel) and by both graft and significantly stenosed native vessel. There is a significant trend that areas supplied by grafts only are more prone to delayed contrast arrival and reduced upslope (table 3). However, differences from native vessels remain small. Therefore, if the coronary and bypass status is unknown, the interpretation of adenosine first pass perfusion may be complicated by delays in contrast arrival. These delays are usually short but reach up to 4 heart beats in sporadic cases, especially if the native vessel is occluded.

### Limitations

The two groups of patients demonstrate significant differences in terms of sex, prevalence of diabetes, ejection fraction and the presence of LGE. However, our group of patients without CAD represents the patient population that is frequently tested to exclude significant CAD and we gain our visual experience in normal perfusion studies. Additionally, a control group of patients with CAD but no significant stenosis is unlikely to have more homogenous perfusion patterns than patients with exclusion of CAD. The more pronounced increase in heart rate during adenosine stress is most probably due to the lesser medication with β-blockers in the patients without CAD (data not shown). The study aimed to compare the semiquantitative parameters in patients without any stenosis in bypasses and unbypassed native vessels. We therefore cannot be certain that the differences found may not be mistaken for real perfusion defects, assuming that the parameters assessed do reflect the visual impression of perfusion. However, these differences are small and can also be found in the patient population without CAD. An additional study may have to address this issue, especially taking into account the possible differences of the endo- and epicardium, as real perfusion defects are mostly non-transmural [19]. When comparing CMR perfusion and coronary angiography, it remains a comparison of functional and anatomical information. Additionally, there is always the possibility of anatomical mismatch when defining perfusion areas of coronaries within the two dimensional X-ray angiography. However, angiography was analyzed by a cardiologist familiar with invasive and perfusion technologies and care was taken not to include segments with uncertainty concerning the perfusing coronary artery or bypass graft. Therefore, not all segments per patient were analyzed. We are, however, certain by including core segments to have the least possible mixture of different vessel territories.

## Conclusions

In conclusion, when comparing semiquantitative perfusion parameters in patients with CABG, there is a small, but not systematic delay in contrast arrival in areas perfused by vessels with bypasses compared to native vessels, especially by the LIMA graft when the native LAD is occluded. However, maximal signal intensity and upslope are not altered and there is no add-up effect of the different parameters. Therefore adenosine stress perfusion studies are most likely not limited in patients post CABG.

## List of abbreviations used

BMI: Body mass index; CABG: Coronary arterial bypass graft; CAD: Coronary artery disease; CMR: Cardiovascular magnetic resonance; FA: Flip angle; LAD: Left anterior descending coronary artery; LCX: Left circumflex coronary artery; LGE: Late gadolinium enhancement; LIMA: Left internal mammary artery; LV: Left ventricle; PET: Positron emission tomography; RCA: Right coronary artery; SI_max_: Maximal signal intensity/peak myocardial enhancement; SSFP: Steady state free precession; TE: Echo time; TR: Repetition time; T_SImax_: Time to 50% of maximal myocardial signal intensity; T_SI50%max_: Time to 50% of maximal myocardial signal intensity.

## Competing interests

Bernhard Schnackenburg is an employee of Philips Medical Systems, Hamburg, Germany.

## Authors' contributions

SK performed CMR image analysis, carried out CMR examinations and drafted the manuscript. KG and SD performed image analysis (invasive angiography). BS conceived the study and participated in the study design. EF conceived the study and helped with the revision of the manuscript. CK designed and coordinated the study, performed CMR image analysis and helped with the revision of the manuscript. All authors have made revisions to the manuscript and have read and approved the final version.
